# Neoadjuvant hormonal therapy is a feasible option in laparoscopic radical prostatectomy

**DOI:** 10.1186/1471-2490-12-36

**Published:** 2012-12-18

**Authors:** Taku Naiki, Noriyasu Kawai, Takehiko Okamura, Daisuke Nagata, Yoshiyuki Kojima, Hidetoshi Akita, Takahiro Yasui, Keiichi Tozawa, Kenjiro Kohri

**Affiliations:** 1 Department of Nephro-urology, Nagoya City University, Graduate School of Medical Sciences, Kawasumi 1, Mizuho-cho, Mizuho-ku, 467-8601, Nagoya, Japan; 2 Department of Urology, East Medical Center Higashi Municipal Hospital City of Nagoya, Nagoya, Japan; 3 Department of Urology, Anjo Kosei Hospital, Anjo, Japan

**Keywords:** Prostate cancer, Neoadjuvant hormonal therapy, Laparoscopic radical prostatectomy

## Abstract

**Background:**

Few reports can be found in the literature with respect to the impact of neoadjuvant hormonal therapy (NHT) on operative parameters on laparoscopic radical prostatectomy (LRP) in a large study. The aim of this study was to evaluate the safety and efficacy of NHT prior to LRP for locally confined prostate cancer.

**Methods:**

From January 2004 to September 2009, 342 patients undergoing LRP were analyzed, specifically comparing 72 patients who received NHT to 270 who did not. All patients were in clinical stage T2 and nerve sparing LRP were not included.

**Results:**

The mean patient age, preoperative prostate specific antigen (PSA), clinical stage, and biopsy Gleason grade were similar for the NHT and the non-NHT LRP groups. The median blood loss and the median operative time were also similar. There were no differences in the intraoperative complication rate of rectum injury, blood transfusion, and open surgery conversion. The positive surgical margin rate was significantly improved in NHT patients. Moreover, PSA recurrence within two years was significantly less in long-term NHT than in non-NHT patients.

**Conclusions:**

LRP was shown as a safe and efficacious procedure in patients who have received NHT. Perioperative morbidity of NHT patients undergoing LRP appears equivalent to non-NHT patients, with lower positive surgical margin, and PSA recurrence rate.

## Background

In recent years, significant improvements have been made in the early detection of prostate cancer (PCA). Also, a rapid increase in incidence during the past two decades has been noted [1]. Radical retropubic prostatectomy (RP), in particular, provides excellent long-term disease control for patients with clinically localized PCA [2]. Laparoscopic prostatectomy (LRP), first described by Schuessler et al. [3], is a standard treatment modality for localized PCA that seeks to combine the benefits of a minimally invasive approach with the advantages of surgical removal and pathologic staging of the tumor. This technique was initiated in our practice in 2001, and since then more than 500 cases have been experienced.

The use of neoadjuvant hormonal therapy (NHT) lacks widespread acceptance in the treatment of PCA patients. Studies have demonstrated a decrease in pathologic stage without improvement in prostate specific antigen (PSA) for disease-free survival in patients receiving 3 months of NHT [3-5]. In contrast, recent studies [6,7] have shown the effectiveness of NHT was enhanced by using it for a longer duration (>8 months) or combining it with androgen blockade. Prostatic apoptosis associated with prostatic and periprostatic fibrosis has been seen after NHT. A consensus has not been possible on whether or not these periprostatic changes make RP more difficult because of insufficient data on this question. To our knowledge, few reports can be found in the literature with respect to the impact of NHT on operative parameters on LRP in a large study. However, one study [8] reported a decrease in seminal vesicle invasion rate with NHT. Therefore, the aim of this retrospective study was to compare the results of LRP in patients who did and did not receive NHT prior to LRP especially in high risk PCA patients.

## Methods

### Study population

Between January 2004 and September 2009, 342 men were scheduled for LRP as the treatment for apparently localized PCA. LRP was performed as described by Guillonneau and Vallancien [9]. After removing the prostate, the specimen was fixed in 10% buffered formaldehyde. After removal of the apex and the bladder neck resection margins, the prostate was sectioned axially at regular intervals of 5 mm or less, yielding serial slices of tissue. On each slide, a pathologist outlined the region of cancer and assigned a Gleason grade. Clinical characteristics are listed in Table 1. Of the 342 patients, 72 had received NHT. In the NHT group 50 patients were treated with anti-androgen and luteinizing hormone-releasing hormone analogue. The other 22 patients were treated only with anti-androgen alone. The period of NHT was 3.8 (0.5-24) months prior to LRP. NHT was initiated due to the concerns of the patients to delay the tumor progression while waiting for the operation to be scheduled. Nerve sparing cases were omitted. Preoperative and perioperative clinical and pathological data were recorded including patient age, preoperative PSA, biopsy Gleason grade, clinical stage, pathologic stage, blood loss including urine, intraoperative complications and surgical margin status. There were no significant differences among the two groups in clinical characteristics. The oncologic results were evaluated by staging of the operative specimen according to the TNM 2002 classification and the last serum PSA level after operation. PSA recurrence was defined as two consecutive increases > 0.2 ng/mL. The median follow-up for biochemical recurrence free patients was 4.5 (2.0-7.5) years. This study was approved by the institutional review board at Nagoya City University Hospital and conducted in accordance with Declaration of Helsinki. Surgical complications were monitored according to the Clavien-Dindo Classification. Numerical parameters between the two groups were compared using the Student’s *t* test or Man-Whitney *U* test when appropriate. A *P* value less than 0.05 was considered significant.

**Table 1:** Clinical characteristics of laparoscopic radical prostatectomy cases treated with or without neoadjuvant hormonal therapy groups *p **< 0.05**

**Variables**	LRP* alone	LRP with NHT**
No. of patients	270	72
Mean age ± SD	66.3 ± 6.1	67.7 ± 5.4
Mean serum PSA ± SD, ng/mL	8.64 ± 5.2	9.81 ± 4.1
Biopsy Gleason grade, n(%)		
≤ 3 + 3	121 (44.8)	30 (41.7)
3 + 4, 4 + 3	85 (31.5)	25 (34.7)
≥ 4 + 4	64 (23.7)	17 (23.6)
Clinical stage, n(%)		
cT1c	100 (37.0)	27 (37.5)
cT2	170 (63.0)	45 (62.5)
D’Amico risk classification, n(%)		
low	76 (28.1)	14 (19.4)
intermediate	74 (27.4)	24 (33.3)
high	120 (44.4)	34 (47.2)
Pathological stage, n(%)		
pT2	193 (71.5)	62 (86.1)
pT3	77 (28.5)	*10 (13.9)
Median operative time, min	260 (121–572)	276 (151–454)
Median blood loss, ml	600 (50–4500)	600 (90–3928)
Intraoperative complication		
Rectum injury, n(%)	4 (1.48)	2 (2.78)
Blood transfusion, n(%)	4 (1.48)	2 (2.78)
Open surgery conversion, n(%)	10 (3.70)	3 (4.17)
Positive surgical margin, n(%)	114 (42.2)	*20 (27.8)
Biochemical recurrence, n(%)	48 (17.8)	14 (19.4)

Patient characteristics and outcomes in laparoscopic prostatectomy (LRP) only and LRP with neoadjuvant hormone therapy (NHT) *p < 0.05.

LRP*: laparoscopic radical prostatectomy NHT**: neoadjuvant hormonal therapy.

## Results

### Patient characteristics

The clinical features of the patients are shown in Table 1 as indicated previously. Mean patient age ± standard deviation (SD) was 66.3 ± 6.1, and 67.7 ± 5.4 for the LRP alone group and LRP with NHT groups respectively. Mean serum levels of PSA before prostate biopsy and prostate biopsy Gleason scores were similar in the two groups. About 37% of each group was in clinical stage cT1c. The two groups were equally balanced for clinical characteristics.

### Influence on operation and postoperative parameters

There were no differences in median operative times (260 min in LRP alone, and 276 min in LRP with NHT), and in median blood loss (600 ml in LRP alone, and 600 ml in LRP with NHT). With respect to the intraoperative complications, no differences were seen in the rate of rectum injury, open surgery conversion, and the necessity of blood transfusion. All complications were less than grade 1 in the Clavien–Dindo Classification, and were treated as routine procedures. There was also no difference in the complication rate between the two groups on the history of abdominal surgery. Several patients had experienced an appendectomy or total gastrectomy, but had no effect on the complication rate in such patients (data not shown). There was no mortality in this series. Positive surgical margin (PSM) was demonstrated in 114 (42.2%) in the LRP alone group and in 20 (27.8%) in LRP with NHT group. The difference between these two groups was significant. The apex was the most common location of PSM in each group (data not shown).

### Influence on biochemical recurrence free survival rate

The PSM rate was significantly lower in patients that were treated with NHT. In all, 62 patients had biochemical recurrence (BCR) (48 in LRP alone and 14 in LRP with NHT). The 2-year BCR free probability was similar between the two groups. For further analysis of prolonged NHT, the LRP with NHT group was divided into two groups of less than 3 months (Group A) and more than 3 months (Group B). The clinical features of the patients are shown in Table 2. No clinical differences emerged from studies of the two groups. The comparisons of median blood loss and operative time, and the rate of rectum injury, open surgery conversion, and necessity of transfusion were all not significant. In this analysis, the PSM rate was similar between the two groups, but the 2-year BCR rate was significantly lower in group B than in group A: (13 patients (27.7%) in Group A, versus one patient (4.00%) in Group B).

**Table 2:** Comparison of neoadjuvant hormonal therapy period treated with laparoscopic radical prostatectomy patients

**Variables**	Group A*	Group B**
No. of patients	47	25
Mean age ± SD	67.4 ± 5.6	68.3 ± 4.7
Preoperative serum PSA ± SD, ng/mL	10.0 ± 3.9	9.38 ± 4.4
Biopsy Gleason grade, n(%)		
≤ 3 + 3	20 (42.6)	10 (40.0)
3 + 4, 4 + 3	20 (42.6)	5 (20.0)
≥ 4 + 4	7 (14.9)	10 (40.0)
D’Amico risk classification, n(%)		
low	11 (23.4)	3 (12.0)
intermediate	17 (36.2)	7 (28.0)
high	19 (40.4)	15 (60.0)
Kind of neoadjuvant hormonal therapy		
CAB	32 (68.1)	18 (72.0)
anti-androgen alone	15 (31.9)	7 (28.0)
Pathological stage, n(%)		
pT2	42 (89.4)	20 (80.0)
pT3a	3 (6.40)	4 (16.0)
pT3b	2 (4.30)	1 (4.00)
Median operative time, min	286 (151–454)	270 (152–410)
Median blood loss, ml	600 (90–3928)	552 (95–1600)
Positive surgical margin, n(%)	13 (27.7)	7 (28.0)
Biochemical recurrence, n(%)	13 (27.7)	*1 (4.00)

Patient characteristics and outcomes in two NHT patient groups (*Group A*: NHT on LRP for less than 3 months, *Group B*: NHT on LRP for more than 3 months) *p < 0.05.

Group A*: laparoscopic radical prostatectomy cases treated with neoadjuvant hormonal therapy for less than 3 months.

Group B**: laparoscopic radical prostatectomy cases treated with neoadjuvant hormonal therapy for more than 3 months.

*CAB*: combined androgen blockade with anti-androgen and LHRH analogue. *p < 0.05.

*CAB*: combined androgen blockade with anti-androgen and LHRH analogue.

## Discussion

There is no consensus in the medical literature as to whether RP after NHT is of greater, equal, or of lesser difficulty than RP in patients who have not received NHT. Some have stated that NHT decreases the operative parameters and thereby facilitates the surgical procedure [10,11]. Others have reported no differences in operative time, blood loss, transfusion rate, or complication rate in patients who received or did not receive NHT prior to RP [12-14]. Our interest was directed at learning if the inability to palpate the prostate in these cases would make LRP more difficult, especially in the first group of cases that were encountered. In order to focus on this problem, a retrospective study was performed on our large number of patient outcomes. Both patient groups who did and did not receive NHT showed similarities in preoperative clinical features, operative times, blood loss, and intraoperative complication rates. Thus, it appears that LRP is a safe procedure that can be performed easily in both groups of patients.

In this series, the effect of prolonged NHT (over 4 months) was compared to less than 3 months NHT, and no impact was found on operative parameters. Gleave et al. [4] reported a mean blood loss of 761 ml and no major intraoperative morbidity after 8 months of NHT. After 4 months of NHT, Powell et al. [15] found RP was feasible in terms of resectability in patients who earlier might have been considered inoperable due to clinical stage T3/T4 disease. As to LRP, Rassweiler et al. [16] evaluated 180 patients who underwent LRP. Of these, NHT was given in 42 patients (23.3%) that required a longer operative time (321 min) and higher blood transfusion rate (46%) than in patients without NHT. However, in our study, the operative parameters between prolonged NHT and short term NHT were similar. This might be partly due to the recent advances of surgical instruments, for example, clear view system, or superior blood coagulation devices. Nowadays, robot-assisted laparoscopic radical prostatectomy (RALP) is gaining in popularity for the treatment of clinically localized PCA. The benefits of RALP are minimally invasive surgery with wide and 3-dimensional vision and delicate control of instruments. These are considered more reasonable and safe, and constitute an effective treatment modality superior to not only RP but also LRP. Therefore, RALP after NHT might be a feasible option in localized PCA patients.

The BCR free survival is another focus of attention after NHT prior to RP. Meyer et al. [5] reviewed 680 men and follow-up for 38 months after RP. They reported a 33% PSA recurrence rate. The literature is inadequate on the impact of NHT on the outcome of LRP. As to LRP, Pu et al. [6] evaluated 55 patients with clinically localized PCA who were treated with NHT for 3-months (25 patients), and for 8-months (19 patients) and those with non-NHT (11 patients) before LRP. The PSM rate was significantly lower in the 3- or 8-months NHT groups than in the non-adjuvant group (*P* < 0.05, respectively). Also, there was no difference between the 3- and 8-months groups with respect to PSM rate. However, they had no follow-up data as to whether or not prolonged (8-months) NHT prior to LRP altered BCR rates. Table 3 reviews the impact of NHT prior to LRP on PSM and BCR rate [6,7,16-18]. Brown et al. [7] studied the safety and efficacy of LRP after NHT. LRP appeared to be a safe and efficacious procedure. These results were based on 5 patients who received NHT against 60 who did not. They also noted that 2 of 60 patients in the non-NHT group had biochemical recurrence, compared to 0 of 5 patients in the NHT group. This data of a small number of patients and short study term discouraged any statistical study of the cohorts. In contrast, our data includes an extended median follow-up period of 4 years, which strengthens the case for NHT improving BCR free survival. Further large scale prospective investigations are needed.

**Table 3:** Comparison of neoadjuvant hormonal therapy on the outcome of laparoscopic radical prostatectomy

**References**	Period of NHT* (months)	**No. of patients**	**Transfusion rate (%)**	PSM** rate (%)	BCR*** rate after 2 years (%)
Rassweiler et al. [14]					
NHT group	NA	42	46.0	NA	NA
non-NHT group	-	138	NA	NA	NA
Gregori et al. [15]					
NHT group	less than 3	21	NA	NA	NA
non-NHT group	-	59	NA	NA	NA
Brown et al. [7]					
NHT group	less than 3	5	0	0	NA
non-NHT group	-	60	1.7	17.0	NA
Maldonado-Valadez et al. [16]					
NHT group	3.5	50	36.0	18.0	NA
non-NHT group	-	50	52.0	16.0	NA
Pu et al. [6]					
NHT group	3	25	32.0	12.0	NA
	8	19	31.2	10.5	NA
non-NHT group	-	11	36.4	45.5	NA
Our series					
NHT group	3 or less	47	2.12	27.7	27.7
	more than 3	25	4.00	28.0	4.00
non-NHT group	-	270	1.48	42.2	17.8

Reviews of the impact of NHT prior to LRP on operative and postoperative parameters.

NHT*: neoadjuvant hormonal therapy PSM**: positive surgical margin BCR***: biochemical recurrence.

*NA*: not available *PSM*: positive surgical margin *BCR*: biochemical recurrence.

Several limitations and factors might account for the lack of difference in BCR rates despite improved PSM rates between the non-NHT and NHT groups. In the present study about 25% were low risk patients according to the D’Amico classification (PSA < 10 ng/mL, biopsy Gleason score < 7, cT1) with an associated low risk of BCR after RP alone. Such patients might derive little benefit from the addition of NHT. Similar to other previous studies, this trial was also initially designed to identify differences in pathological stage and not powered to detect differences in BCR. NHT causes shrinkage and condensation of the benign prostate tissue around the tumor. Thus, while the apoptosis caused by NHT may increase the number of apparently negative margins, tumors may actually be closer to the prostate capsule, margin, and seminal vesicle. The lack of a demonstrable BCR benefit for NHT in a long term follow-up might be due to insufficient sample size. In a meta-analysis that combined several studies to include 1129 men, NHT did not improve BCR rates after RP. In another study of 156 patients treated with 8 months of NHT, Gleave et al. [19] reported low PSM (12%) and BCR (12.2%) rates at a mean follow up of 54 months. In our study, there was no difference in preoperative risk between the two groups (LRP alone vs. LRP with NHT) in Table 1. However, the number of pathological stage T3 patients was significantly lower in the NHT group. Table 2 shows the high risk PCA patients tended to be higher in the long-term NHT group, but the BCR rate was significantly lower. Although this was a retrospective study, a longer NHT period might decrease the active capsular penetration and seminal vesicle invasion, and as a result prevent the biochemical recurrence. Our findings suggest that the optimal duration of NHT on LRP might be over 3 months similar to RP.

In Japan, there are a limited number of large institutes where LRP can be performed. A Japanese preoperative nomogram was reported which indicated the probability of extracapsular penetration after surgery is 15-27% in low risk patients [20]. Patients were informed of the probability and the risk of comorbidity before surgery. When the patient chose the NHT, it was performed according to the waiting time for the operation. There was no comorbidity in long-term NHT group. To truly assess the appropriateness of NHT requires a careful consideration of the growing evidence of risk of the cardiac and metabolic diseases associated with such long-term exposure to androgen deprivation. Consequently, many diagnosed cases of PCA have a long waiting period before surgery can be scheduled. Thus, many cases are treated with NHT to suppress the progression of the malignancy. Our data showed that LRP is safe and effective for the treatment of PCA in men who received NHT. The longer period of NHT can reduce the PSM, and might improve the BCR free survival on LRP.

## Conclusion

LRP was shown as a safe and efficacious procedure in patients who have received NHT. Perioperative morbidity of NHT patients undergoing LRP appears equivalent to non-NHT patients, with lower positive surgical margin, and PSA recurrence rate.

## Competing interest

The authors declare that they have no competing interests.

## Authors’ contributions

This manuscript has not been published elsewhere in part or in entirety and is not under consideration by another journal. Further, all the authors have read and approved the manuscript and agree with its submission to your journal. Details regarding authorship, conflicts of interest, and ethical approval are given in the accompanying Author Submission Requirement Form. Each author participated sufficiently in the work to take public responsibility for appropriate portions of the content. TN carried out to design the study and make statistical analysis, and drafting of the manuscript. NK made critical revision of the manuscript. TO and DN carried out the acquisition of data. YK and HA participated in the design of the study and performed the statistical analysis. TY and KT participated in its design and coordination and helped to draft the manuscript. KK made supervision of this study. All authors read and approved the final manuscript.

## Pre-publication history

The pre-publication history for this paper can be accessed here:

http://www.biomedcentral.com/1471-2490/12/36/prepub
